# Human Gingiva-Derived Mesenchymal Stem Cells Inhibit Xeno-Graft-versus-Host Disease *via* CD39–CD73–Adenosine and IDO Signals

**DOI:** 10.3389/fimmu.2017.00068

**Published:** 2017-02-02

**Authors:** Feng Huang, Maogen Chen, Weiqian Chen, Jian Gu, Jia Yuan, Yaoqiu Xue, Junlong Dang, Wenru Su, Julie Wang, Homayoun H. Zadeh, Xiaoshun He, Limin Rong, Nancy Olsen, Song Guo Zheng

**Affiliations:** ^1^Department of Clinical Immunology, Third Affiliated Hospital at Sun Yat-sen University, Guangzhou, China; ^2^Organ Transplant Center, First Affiliated Hospital at Sun Yat-sen University, Guangzhou, China; ^3^Division of Rheumatology, Penn State Hershey College of Medicine, Hershey, PA, USA; ^4^Division of Periodontology, Diagnostic Sciences and Dental Hygiene, University of Southern California Ostrow School of Dentistry, Los Angeles, CA, USA

**Keywords:** gingival mesenchymal stem cell, GVHD, indoleamine 2,3-dioxygenase, CD39, CD73, adenosine, immune regulation

## Abstract

Mesenchymal stem cells have the capacity to maintain immune homeostasis and prevent autoimmunity. We recently reported that human-derived gingival mesenchymal stem cells (GMSCs) have strong capacity to suppress immune responses and T cell-mediated collagen-induced arthritis in animals. However, it is unclear whether these cells can suppress human T cell-mediated diseases. Here, we used a xenogenic GVHD model in the NOD/SCID mouse, which is a useful preclinical construct for evaluating the therapeutic and translational potential of this approach for applications in human disease. We found that GMSCs potently suppressed the proliferation of PBMC and T cells *in vitro*. Co-transfer of GMSC with human PBMC significantly suppressed human cell engraftment and markedly prolonged the mouse survival. Moreover, we demonstrated that GMSCs inhibited human PBMC-initiated xenogenic responses *via* CD39/CD73/adenosine and IDO signals. These findings suggest the potential for GMSCs to suppress human immune responses in immune system-mediated diseases, offering a potential clinical option to be used for modulating GVHD and autoimmune diseases.

## Introduction

Allogenic graft-versus-host disease (allo-GVHD) is a severe complication of organ or bone marrow (BM) transplantation, which causes significant morbidity and mortality (1). The current strategies to treat allo-GVHD usually fail to achieve a long-lasting response (2, 3).

The successful engraftment of human cells to mouse (human to immunodeficient mouse chimera) also results in xenogenic GVHD (xeno-GVHD) that largely leads to fatal syndromes in NOD/SCID mice (4). Xeno-GVHD model provides an ideal preclinical tool to study the function and efficacy of human cells *in vivo* prior to studies in humans.

Mesenchymal stem cells (MSCs) are stromal cell progenitors with the ability to differentiate down mesodermal cell lineages (5). They also have wide-ranging immunomodulatory properties that contribute to anti-inflammatory effects (6–8). Most frequently investigated MSCs are those derived from BM, adipose, and umbilical tissues (9, 10). Nonetheless, each MSC population has weaknesses. For example, BM-MSCs from patients with autoimmune diseases are dysfunctional, limiting the use of autologous MSCs for disease treatment. Use of BM-MSCs with long term has a potential risk on tumorigenesis (11–13) and did not achieve effective results for disease treatment (14). Invasive procures, pain, and infection risk also restrain the use of BM-MSCs and adipose-MSCs. Very limited cell yields are a weakness for use of umbilical tissue-derived MSCs.

We and others have recently reported that human gingival tissue-derived MSCs (GMSCs) shared similar phenotypic and functional characteristics as other MSCs in multiple animal experimental models, including colitis, collagen-induced arthritis, wound healing, and contact hypersensitivity (15–18). However, the effect of human GMSCs on human immune cell-mediated diseases has not been previously reported.

In this paper, we present evidence that adoptive transfer of human GMSC markedly inhibits the development of xeno-GVHD through a mechanism involving indoleamine 2,3-dioxygenase (IDO) and CD39/CD73/adenosine signals. The results suggest that manipulation of GSMC provides a promising approach to treatment of human immunological diseases.

## Materials and Methods

### Animals

NOD/SCID mice were purchased from the Jackson Laboratory. This study was carried out in accordance with the recommendations of the Institutional Animal Care and Use Committees (IACUC) approved by at the Sun Yat-sen University, the University of Southern California and the Penn State University Hershey Medical Center.

### Gingiva-Derived MSCs

Human gingiva samples were collected following routine dental procedures at the Division of Dentistry in the Third Hospital at Sun Yat-sen University and the School of Dentistry of USC, which were approved by the medical ethics committees of Institutional Review Boards (IRB) at the Third Hospital at the Sun Yat-sen University and the Keck School of Medicine at the University of Southern California and at the Penn State University Hershey Medical Center. The written informed consent was obtained from the donors. The transfer of gingival tissues from the University of Southern California to the Penn State University Hershey Medical Center was approved by both institutions. Human GMSCs were prepared from these samples as previously described (15, 19). BMSCs were obtained from ATCC (PCS-500-012).

### Immune Cell Isolation and Preparation

CD3^+^ T cells were prepared from human PBMCs as described before (20). Non T cells were preserved and irradiated with 30 cGy using a ^137^Cs source and served as antigen-presenting cells. CD4^+^CD25^+^ nTreg cells were acquired from T cells by cell sorting, gating on CD4^+^CD25^bright^CD127^−^ cell population, with >99% purity of the selected population.

### GMSCs Immunosuppression Assays

GMSC or GMSCs pretreated with indicated reagents were cocultured with CD3^+^ T cells, ratio of GMSC to T cells was 1:5. Different reagents, including selective heme oxygenase1 (HO-1) inhibitor [50 ng/ml zinc protoporphyrin IX (ZnPP); Frontier Scientific], HO-1 inducer (50 ng/ml hemin; Sigma-Aldrich), nitric oxide (NO) synthase inhibitor [1 mM l-NG nitroarginine methyl ester hydrochloride (l-NAME); Sigma-Aldrich], indoleamine 2,3-dioxygenase (IDO) inhibitor [500 μM l-1-methyltryptophan (1-MT); Sigma-Aldrich], CD73 inhibitor [100–500 µM α,β-methylene ADP (APCP); Sigma-Aldrich], a CD39 inhibitor [100 µM sodium polyoxot-ungstate 1 (POM1); Tocris Bioscience], selective cyclooxygenase 1 (COX-1) inhibitor (20 µM indomethacine; Sigma-Aldrich), selective COX-2 inhibitor (10 µM NS398; Tocris Bioscience), ALK5 inhibitor (10 µM), anti-transforming growth factor β (anti-TGF-β) antibody (10 µg/ml; BD PharMingen), selective A2B adenosine receptor antagonist (10 µM alloxazine; Sigma-Aldrich), selective A2A adenosine receptor competitive antagonist (25 µM SCH58261; Tocris Bioscience), were added to the coculture system, and the suppression rate was determined by Flow Cytometry. To exclude the non-specific role of these inhibitors to T responder cells, we have added various inhibitors to baseline alone and GMSC group was compared to each baseline. In other experiments, PBMCs were stimulated with lipopolysaccharides (LPS) (100 ng/ml) with a ratio of PBMCs to GMSCs at 1:5. After 3 days, intracellular cytokines including IL-4, IL-17, and IFN-γ positive cells were determined in CD4^+^ T cells by FACS (21).

### Development of Mouse Xeno-GVHD Model

Mice were irradiated by 2.5 cGy TBI using a ^137^Cs source 2–4 h before PBMC injection. CD25-depleted PBMC (20 × 10^6^) were injected intravenously into mice *via* tail vein. Four hours later, PBS, GMSCs, human Fibroblasts, or nTregs were intravenously co-transfused. Mice were monitored every 2–3 days for weight loss and assessed for symptoms of GVHD. Blood samples were taken weekly to detect the percentages of human CD3^+^ T cells and cytokine production and Foxp3 expression among CD4^+^ cells. Serum samples were used to measure cytokines and antibodies by ELISA. At the terminal point of observation, mice were sacrificed for cytokine measurements and histologic analysis. To determine the underlying mechanisms, a tryptophan analog 1-Methyl-d-tryptophan (1-MT) (Sigma) was administered i.p. at 10 mg/mouse/day for 14 consecutive days. Additionally, GMSCs cells were pretreated with POM-1 (Tocris Bioscience; 100 µM) overnight before being injected into mice.

### Statistical Analysis

Data are represented as mean ± SEM. Multiple regression and Student’s *t*-test were used for statistical analysis. The mouse survival was determined by the Kaplan–Meier log-rank test. *p* < 0.05 was considered to be significant.

## Results

### GMSCs Suppress Human Immune Responses through CD39/73/Adenosine and IDO Signals but Independent of Foxp3^+^ Treg Cells *In Vitro*

**Figure 1:**
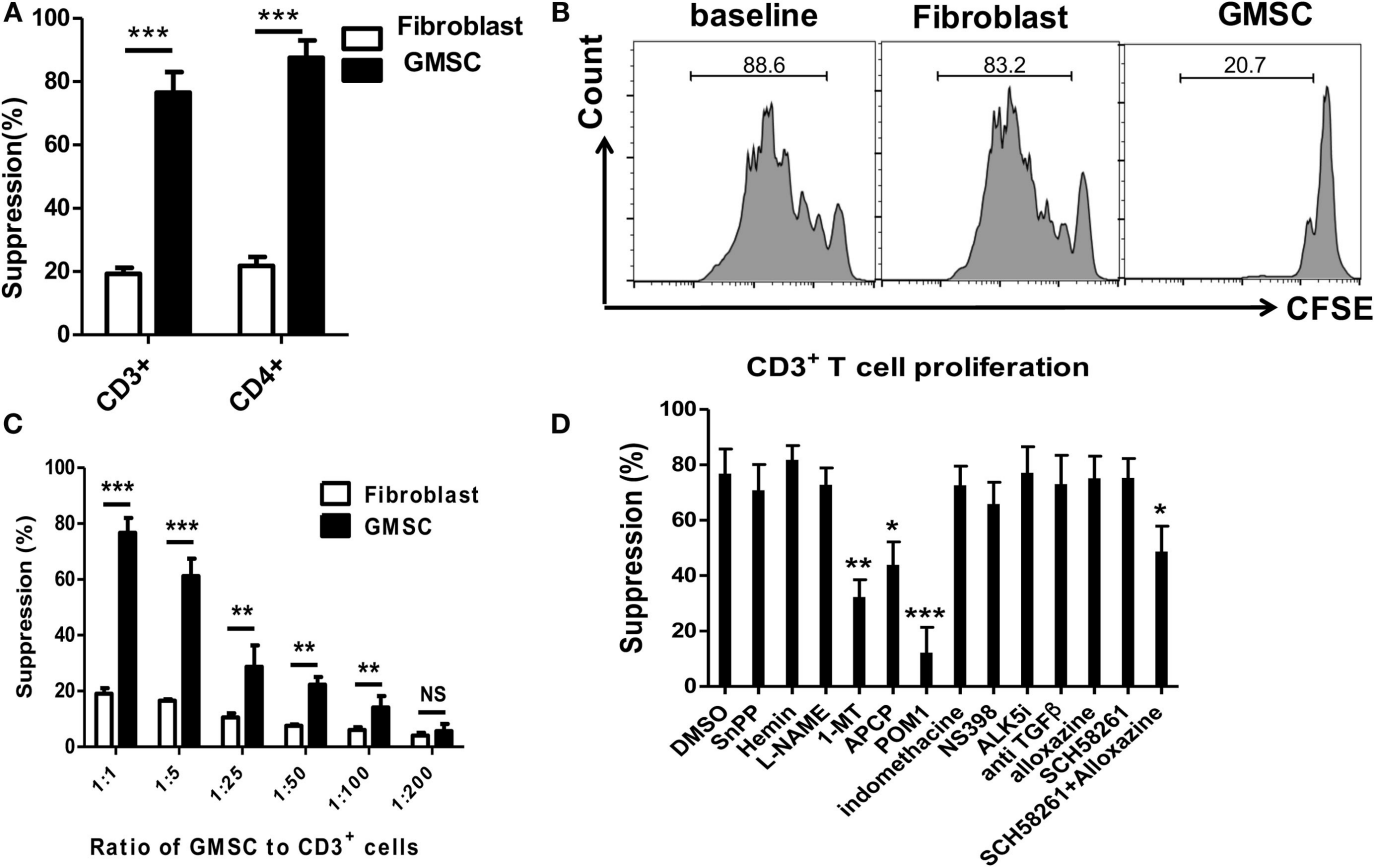
**GMSCs inhibit T cell proliferation through CD39/CD73 and IDO signals**. **(A,B)** Human CD3^+^ T cells were prepared from PBMC and labeled with CFSE. T cells were then stimulated with irradiated non T cells (1:4) and anti-CD3 (1 µg/ml) for 4 days. Human fibroblast cells were used as a negative control. The T cell proliferation was determined by Flow cytometry. A representative experiment is shown in panel **(B)**. **(C)** GMSCs were cocultured with CD3^+^ T cells as described above. Graded doses of GMSCs were added to T cells for 4 days. **(D)** GMSC were cocultured with CD3^+^ T cells, ratio of GMSC to T cells was 1:5. Different reagents as described above were added to the coculture system, and the suppression rate was determined by Flow cytometry. The same ratio of GMSC and reagents were added as in panels **(C,D)**. CFSE dilution was determined by FACS. Data are summarized from three replicate experiments, represented as mean ± SEM. **p < 0.01, ***p < 0.001 versus the own control group containing each inhibitor in baseline well.

We have previously shown that human GMSCs display the typical morphologic and phenotypic characteristics of MSCs and suppress mouse immune responses (19). Now, we demonstrate that GMSCs are able to suppress both human CD3^+^ and CD4^+^ T cells proliferation *in vitro*, while the control cells, human fibroblasts, did not display such suppressive ability (Figures 1A,B). The ratio of 1:1 of GMSCs to T cells resulted in 80% suppression of T cell proliferation, and the suppression appeared to have a dose-dependent effect (Figure 1C). Unlike other MSCs that require HO-1, iNOS, TGF-β, or PGE2 to suppress immune responses (7, 22), unexpectedly, we found that GMSCs inhibited human lymphocytes proliferation through CD39/CD73/adenosine and/or IDO signals *in vitro* (Figure 1D). Since various inhibitors and antagonists possibly directly alter T cell proliferation or indirectly through non-specific role change T cell proliferation, we have added each inhibitor or antagonist to T cell proliferation alone to exclude these roles. We observed that addition of 100 µM CD39 inhibitor markedly abolished the suppressive effect of GMSCs on T cell proliferation (Figure 1D); this dose of CD73 inhibitor did not abolish until its doses were raised to 200–500 µM. It is likely that both inhibitors have a different sensitivity. We also revealed that either A2A or A2B adenosine receptor antagonist did not change GMSCs function; nonetheless, when the both adenosine receptor antagonists were combined, it significantly revised the inhibitor function of GMSCs to T cells (Figure 1D), thus A2A or A2B can compensate each other. We have pretreated GMSCs and then cocultured these GMSCs with T cells, the results are similarly observed (data not shown), further validating that these chemical inhibitors/antagonists do not directly exert their role in T cells. GMSCs express CD39 and CD73 and both are crucial for the degradation of ATP, AMP to generate adenosine that binds both A2A and A2B receptors to initiate immune regulatory effect (23), while IDO is only upregulated in the GMSCs cultured in the presence of IFN-γ or activated T cells (18, 19, 22).

**Figure 2:**
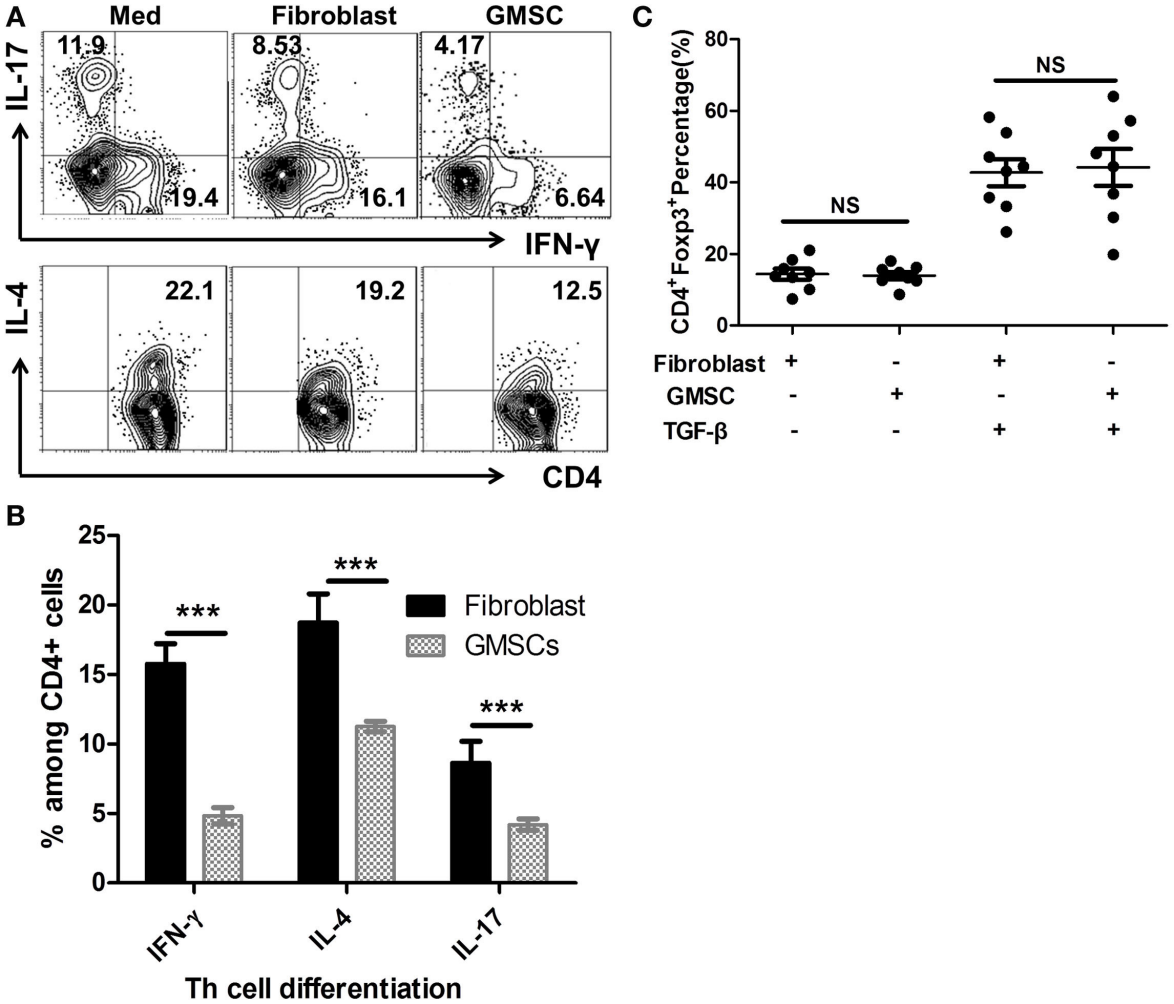
**GMSCs inhibit Th1 and Th17 production, but show no effect on the Foxp3 induction *in vitro***. PBMC were cocultured with GMSC for 3 days under the stimulation of lipopolysaccharides (100 ng/ml) with a GMSC to PBMC ratio of 1:5. Intracellular cytokines IL-4, IL-17, IFN-γ among CD4^+^ cells were analyzed by flow cytometry. Shown are representative flow data **(A)** and frequencies of each cytokine on the gate of CD4^+^ cells **(B)**. Experiments were repeated three times. **(C)** Human naïve CD4^+^ T cells were cocultured with or without GMSC or human fibroblast cells (1:5) and stimulated with anti-CD3/CD28 microbeads (beads: T cells ratio = 1:5) with or without TGF-β, IL-2 for 5 days. Foxp3 expression on CD4^+^ T cells was analyzed by flow cytometry. Data are presented as the mean ± SEM from three independent experiments.

It has been reported that Toll-like receptor 4, which is a major receptor for LPS, was indispensable in the pathogenesis of GVHD (24), and LPS stimulation results in the release of pro-inflammatory cytokines from human peripheral cells (25). We, therefore, analyzed the inflammatory cytokine production following LPS stimulation and found that GMSCs markedly inhibited the secretion of IFN-γ, IL-4, and IL-17 by lymphocytes in this condition (Figures 2A,B). The production of pro-inflammatory cytokines was one of the important mechanisms involved in the pathogenesis of GVHD (3), providing further evidence that GMSCs may suppress GVHD. Furthermore, CD4^+^Foxp3^+^ Treg cells have a potent immunoregulatory role and can suppress GVHD (4, 26–28), so we also sought to determine whether GMSCs can suppress GVHD through Foxp3 upregulation. However, GMSCs did not change Foxp3 expression in CD4^+^ T cells irrespective of the presence of TGF-β *in vitro* (Figure 2C).

### GMSCs Suppress Xeno-GVHD *In Vivo*

**Figure 3:**
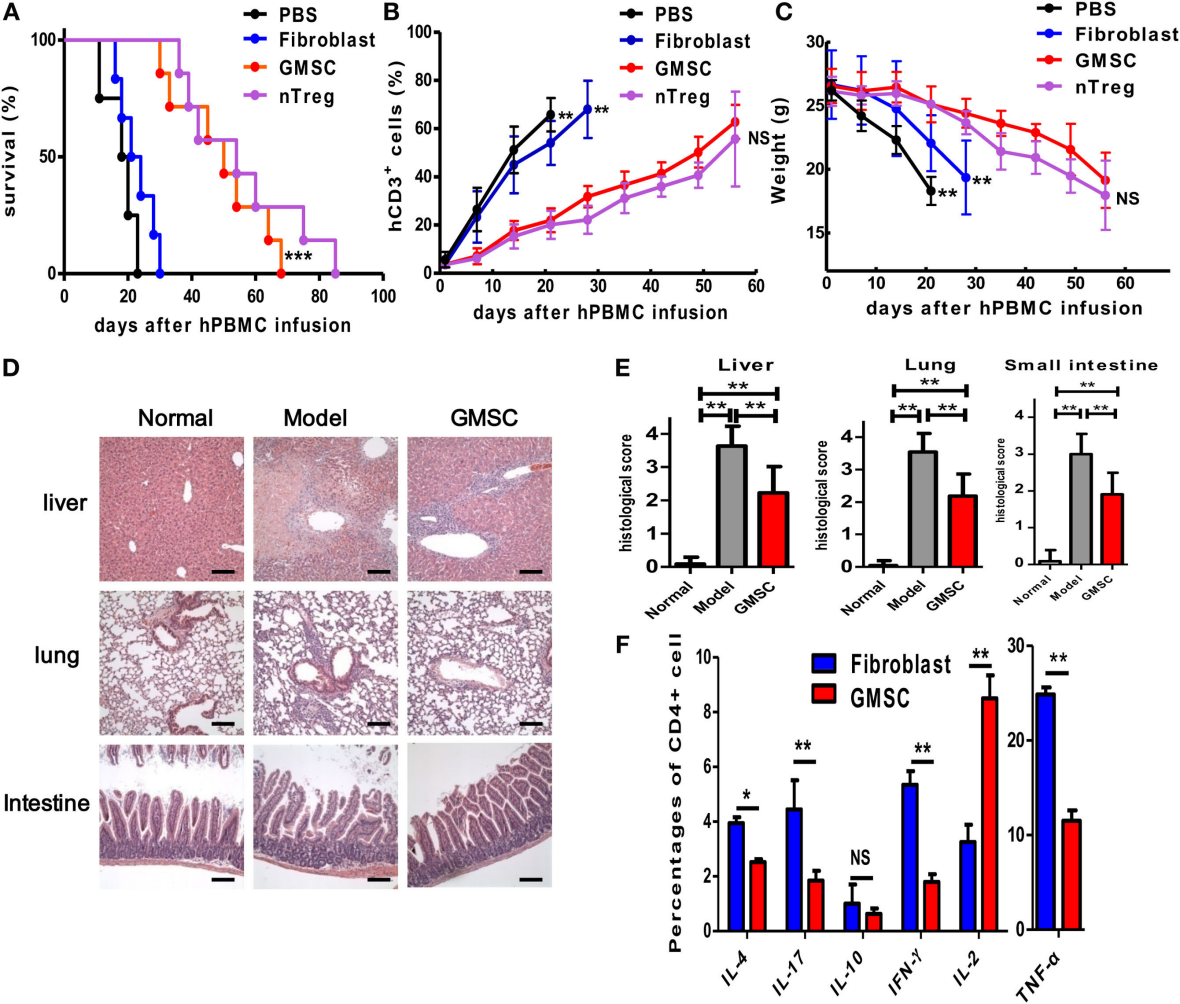
**GMSCs inhibit xenogenic GVHD in NOD/SCID mice**. Freshly isolated human CD25-depleted PBMCs (20 × 10^6^) were injected into NOD/SCID mice intravenously. Mice were co-injected with PBS (*n* = 10), or 4 × 10^6^ *n*Tregs (*n* = 11), or 2 × 10^6^ GMSCs (*n* = 11), or 2 × 10^6^ fibroblasts (*n* = 10). GMSCs were obtained from different donors and used after 2–5 passages. **(A)** Survival of GVHD mice. **(B)** Peripheral blood was collected weekly and stained with anti-human CD3 antibodies and quantified by flow cytometry. **(C)** Average weight is shown as mean ± SEM. **(D)** Liver, lung, and intestine were collected at the time of mouse euthanization and evaluated by hematoxylin and eosin staining for histopathologic investigation. Representative pictures of livers, lungs, and intestines from naive mice model or GMSC-treated model are shown. Scale bars = 200 mm. **(E)** Histological scores were calculated by blinded pathologists and are shown as mean and SEM from groups of 10 mice (*p < 0.05, **p < 0.01). **(F)** When mice were euthanized in the end point of observation, the expression of cytokines by CD4^+^ splenic T cells was determined by intracellular cytokine staining *via* flow cytometry, for IL-2, IL-4, IL-10, IL-17, IFN-γ, and TNF-α. Foxp3 expression was also measured using intracellular flow cytometric analysis. Data are shown as mean ± SEM (*n* = 5 for each group, and experiments were repeated twice). *p < 0.05, **p < 0.01, ***p < 0.001 stands for fibroblast versus GMSC-treated group. NS, not significant.

We, next, analyzed the immunosuppressive capacity of GMSCs *in vivo* in a human to mouse immunodeficient chimera, a xenogeneic GVHD model. As reported previously, transfer of human CD25-depleted PBMC to NOD/SCID mouse results in a typical GVHD syndrome, including weight loss, elevated human pro-inflammatory cytokines, anti-human antibodies, and death of the host animal at around 2 weeks (29, 30). Cotransfer of GMSCs (5 × 10^6^) with CD25-depleted PMBCs (20 × 10^6^) significantly prolonged mouse survival from 15.8 ± 3.76 to 46.0 ± 12.0 days (Figure 3A). Correspondingly, GMSC transfusion resulted in a significantly lower expansion of human T cells in the mouse and significantly prevented weight loss (Figures 3B,C). Furthermore, HE staining demonstrated that the pathological changes and inflammation degrees including necrosis and lymphocyte infiltration present in liver, lung, and intestine were much less in GMSCs-treated mice than in control-treated mice (Figures 3D,E). Accordingly, GMSCs infusion significantly inhibited production by the engrafted human CD4^+^ cells of IFN-γ, IL-4, IL-17, and TNF-α compared to control fibroblasts (Figure 3F). GMSCs did not change IL-10 production and did not suppress IL-2, but rather promoted IL-2 production (Figure 3F). GMSC infusion did not promote Foxp3^+^ cell frequency in donor human CD4^+^ cells (data not shown).

### CD39 and IDO Signals Contribute to the Suppression of GMSCs on Xeno-GVHD *In Vivo*

**Figure 4:**
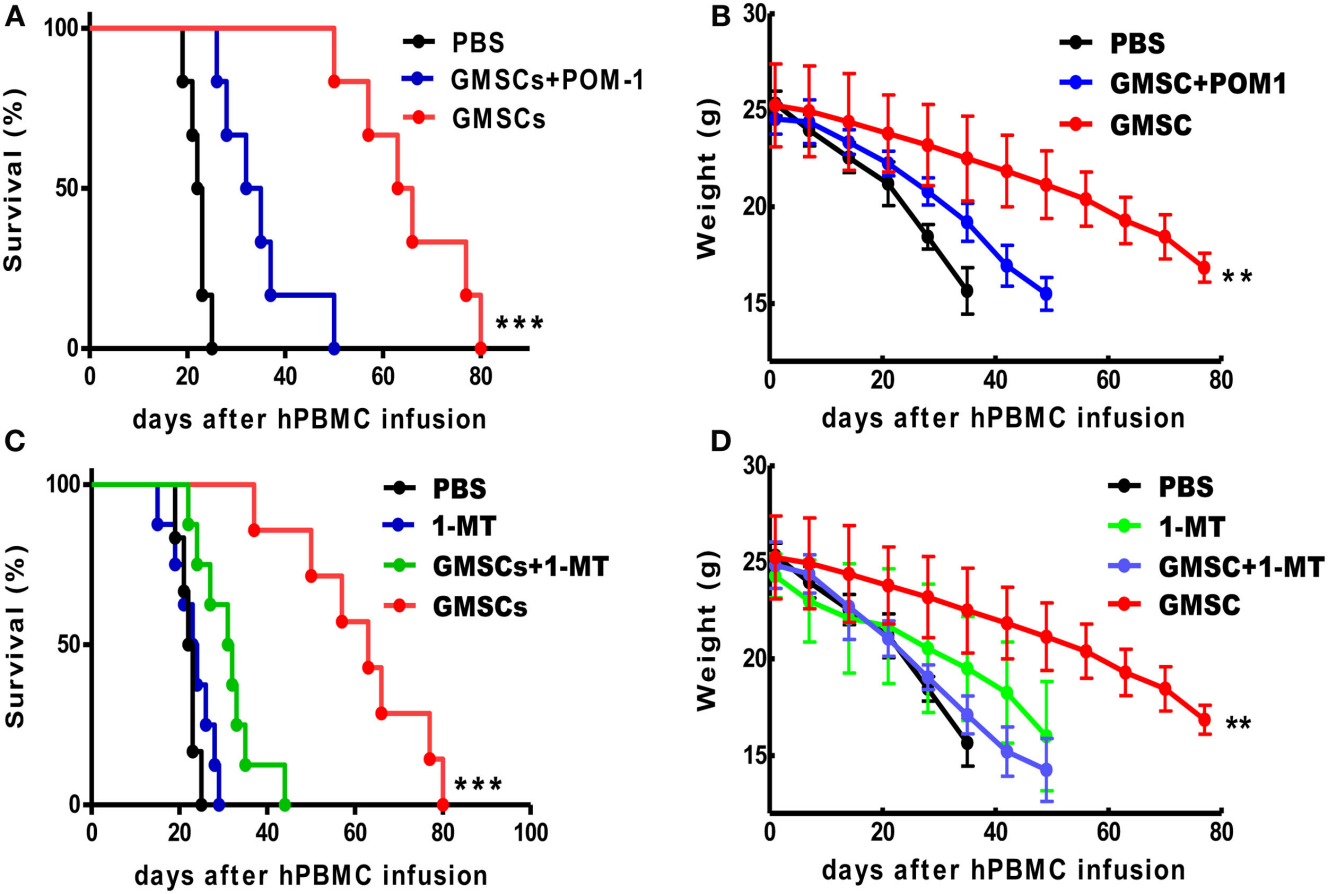
**GMSCs inhibit xenogenic GVHD through CD39 and/or IDO signaling pathways**. NOD/SCID mice were injected with 20 × 10^6^ PBMC with or without 2 × 10^6^ GMSCs (*n* = 8). **(A,B)** Some GMSC cells were pretreated with POM-1 (100 µm) overnight before co-injection with PBMC (*n* = 11). **(C,D)** In another group, mice giving human cell alone or human cells plus GMSCs were injected i.p. with 1-MT at 10 mg/mouse/day for 14 consecutive days (*n* = 10) and then monitored for survival and weight loss. Kaplan–Meier survival curves depict the percentage of live mice. **(B,D)** The weight loss data were presented as the Mean ± SEM from two independent experiments. **p < 0.01, ***p < 0.001 GMSC versus the control treated group.

To identify the mechanism of GMSC function in xeno-GVHD *in vivo*, we analyzed the role of CD39 and IDO signals, since they played a critical role *in vitro* (Figures 1D,E). We selected CD39 blockade since CD39 markedly affects GMSC function *in vitro* and is source of CD39/CD73/adenosine signal pathway. Interestingly, CD39 inhibition with POM-1 or IDO inhibition by 1-MT almost completely abolished the suppressive effect of GMSCs on xeno-GVHD model (Figures 4A,B). We have used continuously i.p. injection of 1-MT but not pretreatment, the key reason is that GMSCs can secrete IDO when they are stimulated with IFN-γ, while this cytokine is rapidly elevated in xeno-GVHD. Moreover, we have adopted the injection of 1-MT to xeno-GVHD model and showed that this administration did not significantly alter the courses and severities of xeno-GVHD (Figures 4C,D). Thus, the effect of 1-MT will be mainly associated with GMSCs. These results highlight the key role of CD39 and IDO in the immunosuppression mediated by GMSCs in human cell immune responses.

### GMSCs Displayed the Superior Effect to BMSCs on Suppressing Xeno-GVHD

**Figure 5:**
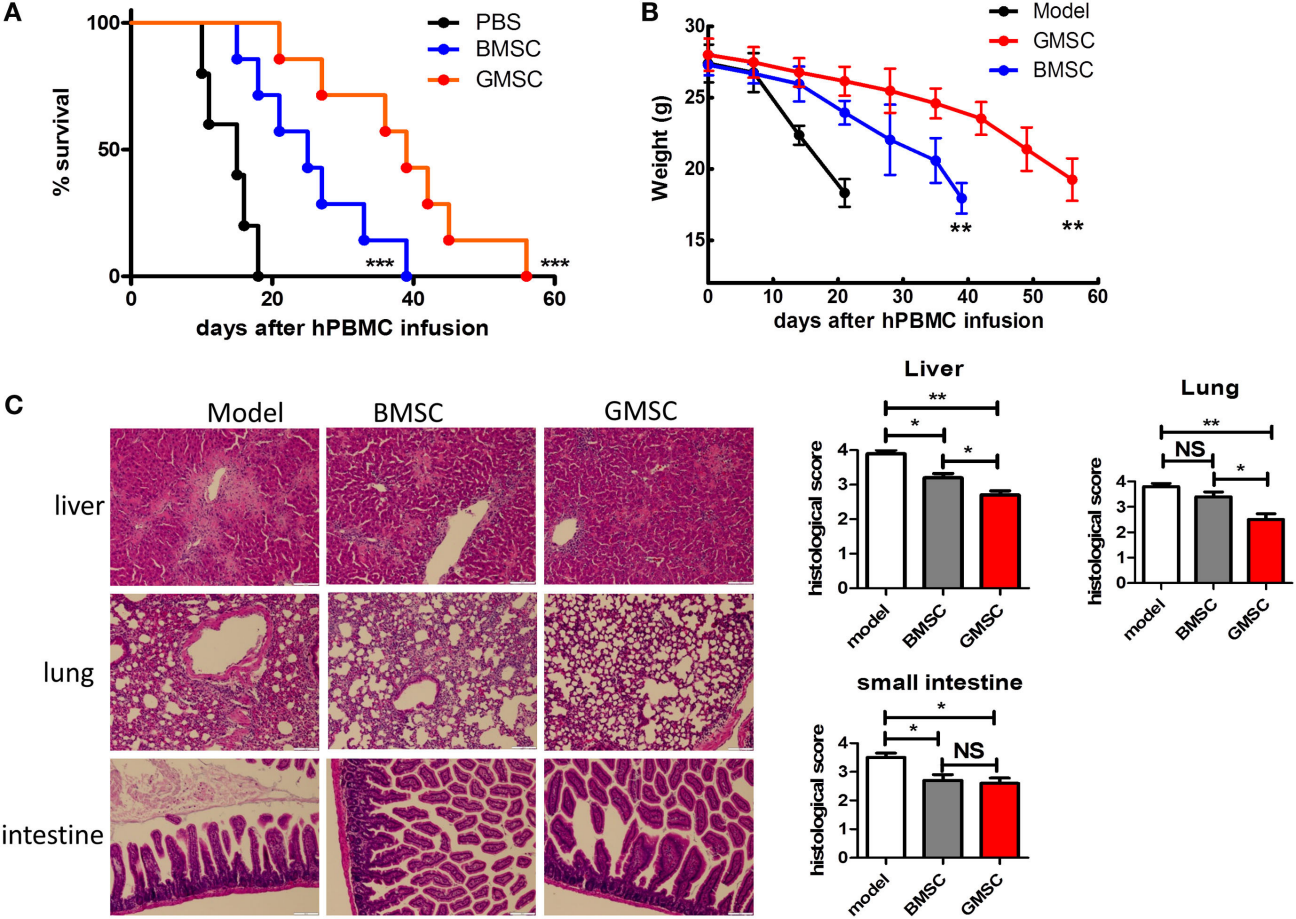
**GMSCs are superior to BMSCs in inhibiting xenogenic GVHD (xeno-GVHD) in NOD/SCID mice**. Freshly isolated human CD25-depleted PBMCs (20 × 10^6^) were injected into NOD/SCID mice intravenously. Mice were co-injected PBMC with PBS (*n* = 10), or 2 × 10^6^ GMSC (*n* = 10), or 2 × 10^6^ BMSC (*n* = 10). GMSCs were obtained from different donors and used after 2–5 passages. **(A)** Survival of GVHD mice. ***p < 0.001 BMSC versus model, or GMSC versus BMSC. **(B)** Average weight is shown as mean ± SEM. **p < 0.01 BMSC versus model, or GMSC versus BMSC. **(C)** Liver, lung, and small intestine were collected at the time of mouse euthanization and evaluated by hematoxylin and eosin staining for histopathologic features. Representative sections of liver, lung, and small intestine from model or BMSC- or GMSC-treated xeno-GVHD were shown (left panel). Histological scores were calculated by blinded pathologists and are shown as mean and SEM from groups of 10 mice. *p < 0.05, **p < 0.01, BMSC versus model or GMSC versus BMSC group. NS, not significant.

Given the existence of differences among the various tissue-derived MSCs, we further compared the functionality of GMSCs and BMSCs in the xeno-GVHD model. We conducted an experiment to directly compare the effect of two MSC populations on xeno-GVHD. The experiments were carried out as described in Figure 3 with similar doses of BMSCs or GMSC cotransferred with human PBMC into NSG mice. As shown (Figure 5), while BMSCs infusion prolonged the survival twofold, GMSC infusion almost prolonged survival threefold. Accordingly, the weight loss and inflammatory pathology in liver, lung, and intestine were much less in GMSC-treated xeno-GVHD than in BMSCs-treated xeno-GVHD, although BMSC treatment did display effects on xeno-GVHD mice.

## Discussion

We present evidence that human GMSCs markedly suppress human immune cell responses *in vitro* and human immune cell-mediated disease progress in a humanized animal xeno-GVHD *in vivo*. To our knowledge, this is first demonstration that human GMSCs can ameliorate human cells-initiated diseases, displaying a potential promise for clinical therapy on autoimmune diseases, GVHD, and prevention of organ transplantation rejection.

We provide insight into potential functional consequences of our finding by identifying CD39/CD73/adenosine signal and IDO pathway as being involved in the regulatory effect of GMSCs. CD39 is an integral membrane protein that phosphohydrolyzes ATP, and less efficiently ADP, in a Ca^2+^- and Mg^2+^-dependent fashion, to yield AMP (31). CD39 is expressed on many human tissues and cells including immune cells that are important for immune homeostasis and function (32). CD39 is highly expressed on MSC, and it is likely that CD39 signal contributes to immunoregulatory effects of MSC (15). Combining with CD73, another ectonucleotidase that degrades AMP to adenosine, CD39 acts as an immune regulatory factor by removing metabolic ATP and producing immune modulating adenosine (33). We previously have shown that CD39 is highly expressed on GMSCs and is needed for their suppressive activity in murine collagen-induced arthritis (19). Given CD39–CD73–adeniosin is a chain to develop immune regulatory molecule, adenosine, it is not surprising that CD73 and adenosine inhibitors also significantly revise the suppressive activity of GMSC. We also observed an interesting phenomenon that adenosine needs both receptors to exert its immuoregulatory role, since blockade of either A2A or A2B receptor did not significantly abolish the suppressive ability of GMSCs.

It is likely that CD39/CD73/adenosine may not be only signal pathway that contributes to immuosuppressive activity of GMSCs. Our findings also indicate that the blockade of IDO in GMSCs significantly abolished the suppression of GMSCs either *in vitro* or *in vivo* in the xeno-GVHD model. It has been recognized that IDO offers an intriguing mechanism for achieving T cell tolerance—it catabolizes the essential amino acid tryptophan, resulting in the generation of a putative pro-apoptotic agent, kynurenine, as well as other downstream immunomodulatory metabolites (7, 34).

IDO is not constitutively expressed by MSC, but can be significantly induced by a variety of inflammatory mediators (29, 31). IFN-γ is one of these inflammatory mediators that is involved in cross-talk between MSC and immune cells. The xeno-GVHD produces a large amount of IFN-γ and TNF-α, which probably in turn stimulate GMSCs to produce IDO (4, 35). Previous study has indicated that GMSCs do not constitutively express IDO, but in response to IFN-γ stimulation, showed a significantly increased level of functional IDO (15). We recently have observed that GMSCs also enhance their immunoregulaotry function following TNF-α stimulation (36). Currently, we do not know what the relationship between CD39 and IDO is, and this is a topic that deserves further study in the future.

It is reasonable that GMSCs suppress the production of multiple cytokines, since these mediators are crucial for disease progression and host mortality. GMSCs displayed significant suppression of Th1, Th2, and Th17 cells in either *in vitro* or *in vivo* conditions. Nonetheless, current study does not rule out whether GMSCs can induce Tr1 or Foxp3^+^ Treg cells. As the NSG recipient lacks immune cells, this model does not permit determination of this possibility. Interestingly, GMSCs promote IL-2 production; detailed mechanisms are unclear, but this has therapeutic implications, since recent studies have indicated low doses of IL-2 can treat several immunological diseases, including GVHD (37, 38).

T regulatory cells are another immunosuppressive factor (39, 40), and some relationship between MSC and Treg cells has been identified (41, 42). We previously have reported that GMSCs can promote the induction of CD39^+^ Foxp3^+^ Treg cells in recipient mouse (43), although GMSCs did not promote Treg induction in the present study that is consistent with another report (44). Further determination of whether in fact Tregs are involved *in vivo* may require a clinical trial of GMSCs in human diseases.

An interesting observation in the current study is the finding that GMSCs are superior to BMSCs in controlling xeno-GVHD. BMSCs are widely used in clinical trials in patients with autoimmune diseases (45–48). Nonetheless, the weaknesses with utilization of BMSCs are significant. First, an invasive approach is required to harvest BM cells. Second, BMSCs from patients with autoimmune diseases are dysfunctional which has limited the adoption of autologous BMSCs for treating diseases. Third, BMSCs are tumorigenic, resulting in a long-term risk of use.

GMSCs express a similar profile of cell surface molecules as BMSC. Additionally, the gingiva is a ready source of material, available in all patients and can be repeatedly sampled if necessary. The cell proliferation *in vitro* is much faster in GMSCs than in BMSCs (15), ensuring the number required for clinical cell therapy. GMSCs are not tumorigenic, an additional advantage for their clinical application. Moreover, we recently observed that GMSCs isolated from active SLE and RA patients shared similar phenotypes and sustained immunoregulatory function similar to healthy controls (Zhang et al., unpublished observation). Thus, it is highly likely that the manipulation of autologous GMSCs represents a potential therapy strategy in human autoimmune diseases, GVHD, organ transplantation, and other immune system disorders.

## Author Contributions

SZ: conception and design, data collection, data analysis and interpretation, manuscript writing, and final approval of manuscript; FH, MC, JG, WS YX, JD and JW: performed experiments, data collection, and data analysis and interpretation; XH and LR: data analysis and interpretation; and HZ and JY: provided gingival tissues. NO: revised and edited the manuscript.

## Conflict of Interest Statement

The authors declare that the research was conducted in the absence of any commercial or financial relationships that could be construed as a potential conflict of interest.
